# Reward-Priming of Location in Visual Search

**DOI:** 10.1371/journal.pone.0103372

**Published:** 2014-07-31

**Authors:** Clayton Hickey, Leonardo Chelazzi, Jan Theeuwes

**Affiliations:** 1 Department of Cognitive Psychology, VU University, Amsterdam, The Netherlands; 2 Center for Mind/Brain Sciences (CIMeC), University of Trento, Trento, Italy; 3 Department of Neurological and Visual Sciences, University of Verona, Verona, Italy; 4 Italian Institute of Neuroscience, Verona, Italy; University of California, Davis, United States of America

## Abstract

Existing visual search research has demonstrated that the receipt of reward will be beneficial for subsequent perceptual and attentional processing of features that have characterized targets, but detrimental for processing of features that have characterized irrelevant distractors. Here we report a similar effect of reward on location. Observers completed a visual search task in which they selected a target, ignored a salient distractor, and received random-magnitude reward for correct performance. Results show that when target selection garnered rewarding outcome attention is subsequently a.) primed to return to the target location, and b.) biased away from the location that was occupied by the salient, task-irrelevant distractor. These results suggest that in addition to priming features, reward acts to guide visual search by priming contextual locations of visual stimuli.

## Introduction

Attention has commonly been characterized as under the control of a.) endogenous, top-down factors, reflecting goal-driven strategy, and b.) exogenous, bottom-up factors, determined by stimulus characteristics and hard-wired sensitivities in early visual cortex [1]–[2]. However, this framework fails to account for a class of findings in the literature that index an endogenous state of the system, but are not strategic in nature [3]. Notable in this regard are results demonstrating the influence of reward history on selective control [4]. Reward appears able to prime vision so that objects with reward-associated features become salient and attention-drawing and this can occur in spite of an observer’s efforts otherwise. For example, we have shown that when a distractor is defined by a color that has recently characterized a rewarded target, it will disrupt target selection even when participants know that the distractor will appear and do their best to ignore it [5]. Anderson, Laurent, and Yantis [6] have similarly found that entrained association of reward to a color will cause distractors characterized by this hue to disrupt search for a unique shape, even when participants are well aware that stimuli color is no longer task relevant, and Kristjánsson, Sigurjónsdóttir and Driver [7] have shown that reward facilitates selection of a target defined by a repeated feature, even when participants are aware that the stimulus is very unlikely to prove rewarding again. Task-irrelevant objects with reward-associated characteristics appear initially well represented in the visual system [5], [8]–[9] before being attentionally suppressed [8], [10], possibly so that the target representation is sheltered from interference [11], [12].

Reward thus creates biases in perceptual and attentional processing that are not indicative of the current goal state of the observer. To date, investigations of this non-strategic influence of reward have focused almost exclusively on representations of low-level visual features and feature-based selection. Results show that objects with reward-associated features or characteristics are preferentially selected regardless of their location [5], [6], [8], [13]–[26]. However, visual search clearly takes place within a spatial coordinate system, and the prior experience of targets and distractors is known to have an impact on how attention is deployed to locations in the future. Here we test the idea that reward might impact the deployment of attention to *locations* in visual search.

The study of location priming in search has a rich history. Seminal work from Rabbitt, Cumming and Vyas [27] demonstrated that correct detection of a set of targets in an array of letters was facilitated when identical target letters were presented at the same position in sequential trials. Treisman [28] extended this finding into the study of feature search, showing that participant response to a target defined by a unique visual feature was faster when target-defining feature and location were both repeated. This suggests that location priming might be contingent on repetition of target-defining features, however Maljkovic and Nakayama [29] later observed that location priming and feature priming could be independently elicited. These authors had participants search for a uniquely coloured shape and discriminate the presence or absence of a notch in one corner of this object, with results showing a benefit for targets that reappeared at the same location and a cost for targets that appeared at a location that had previously held a distractor, regardless of whether the target-defining color was repeated. A critical difference between this study and earlier work is that Maljkovic and Nakayama [29] employed a *compound search paradigm*, in which the response feature is independent of the target-defining feature. This allows one to isolate effects caused by repetition of location from effects caused by repetition of response. Subsequent work using the same paradigm [30] or other types of compound search task [31] have largely reproduced Maljkovic and Nakayama’s [29] findings. Other studies have demonstrated that it is the relative position of a target and distractors that is critical regardless of a change in absolute retinal position [32], suggesting a link between location priming and contextual cueing [33].

In spite of this long interest in location priming in the vision research community, and in spite of the plethora of recent studies investigating the impact of reward on visual features, to our knowledge only 2 existing papers have discussed the impact of reward on location during search. As noted above, Anderson and colleagues [6] used a training task to associate reward to a discrete color, showing that search was disrupted by the presence of distractors characterized by this hue during a subsequent compound search task. Performance in this study was particularly degraded when the target appeared at a location that had held the distractor with reward-associated color in the immediately preceding trial. This suggests that the distractor with reward-associated color drew attention before being strongly suppressed, and that this suppression had a residual impact on the subsequent deployment of attention to the distractor location even when it no longer contained a distractor. While clearly an example of an impact of reward on location, this effect is indirect: it relies on the association of reward to a color. Camara, Manohar and Husain [34] have recently investigated the possibility that reward may have a more direct influence on location. In the dual-task paradigm adopted in this eye-tracking study each trial began with participants moving their eyes to one of two locations identified with circles of identical color. Selection of one of these locations resulted in reward, selection of the other garnered punishment, and participants had no way to determine outcome prior to making the eye movement (see Experiment 2). Following reward feedback participants were required to complete a second visual search task where they made an eye movement to a green target while ignoring a pink distractor. Results showed an increased likelihood that the eyes would be deployed to the pink distractor when it appeared at the location that had garnered reward in the immediately preceding task. Results from this graceful study are thus in line with the idea that reward can prime locations (independent of its impact on features), but aspects of the experimental design leave room for further investigation. Perhaps most importantly, in all experiments reported in this study reward outcome was contingent on the nature of overt participant behaviour. This opens the possibility that reward may have primed the saccadic behaviour rather than the covert deployment of attention or perceptual representation.

**Figure 1:**
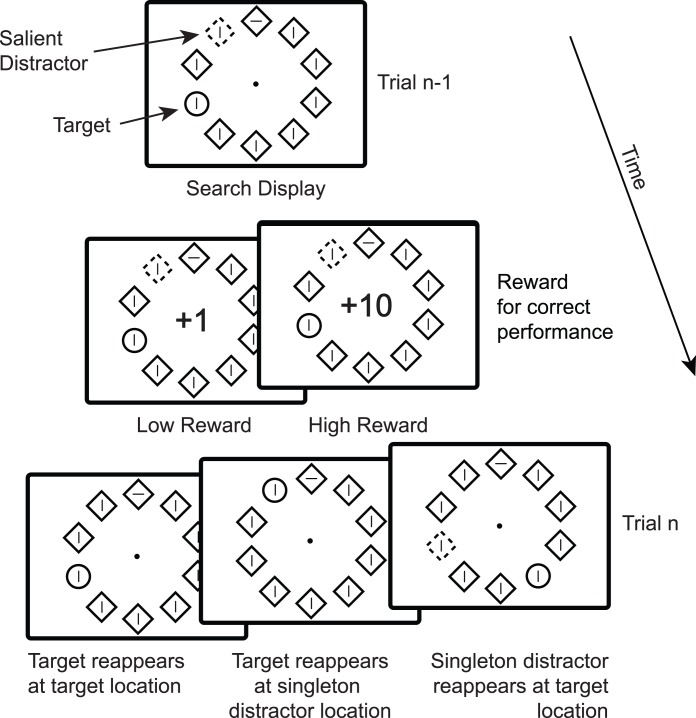
Experimental paradigm.

Here we further investigate the effect of reward on location priming in search. Participants completed a compound visual search task described in earlier papers [5], [18]–[19]. While maintaining eye fixation they were required to covertly select a target defined by unique shape and discriminate the orientation of a line segment contained within it. In many trials they had to ignore a distractor defined by unique color and after each correctly performed trial they received 1 or 10 points (see Figure 1). The number of points thus accumulated determined earnings at the conclusion of the experiment. We analyzed performance on a given trial as a function of a.) the magnitude of point reward received in the preceding trial, and b.) whether target and distractor locations were repeated.

The design has two important characteristics. First, as a compound search task, it decouples the visual feature that defines a target from the visual feature that defines response. As noted above, this allows for repetition effects on perception and selection to be distinguished from repetition effects on response. Second, the magnitude of reward feedback received on any correctly completed trial was randomly determined. There was thus no motivation or opportunity for participants to establish a strategic attentional set for target characteristics like color, form, or location.

We approached the data with the general idea that selective attention relies on both facilitatory mechanisms that act on targets (and their locations) and inhibitory mechanisms that act on distractors (and their locations) [35]–[36]. From this, we generated 4 central experimental hypotheses: reward should: a.) create a benefit when the target reappears at the same location, b.) create a cost when the target appears at the location that previously held the distractor, c.) create a benefit when the distractor reappears at the same location, and d.) create a cost when the distractor appears at the location that previously held the target.

## Method

### Ethics statement

All procedures were approved by the VU University Amsterdam psychology department ethics review board and adhered to the principles detailed in the Declaration of Helsinki. All participants gave written informed consent before participation.

### Summary of approach

To test the hypothesis outlined in the introduction we first reanalyzed existing results from 78 participants who took part in one of a set of three existing experiments (see details below). Each of these experiments was designed to examine the impact of reward on the priming of visual features, an issue that is separate from the possible impact of reward on the priming of locations that is the topic of the current study. The primary result from this reanalysis of existing data was a 3-way interaction in RT. We confirmed this 3-way interaction in a new sample of 17 participants before collapsing across all four experiments to create a 95-person sample. Follow-up statistics designed to identify the specific effects underlying the 3-way interaction were conducted on this large sample. This somewhat complicated approach was adopted for two reasons. First, it provided the opportunity to confirm the 3-way interaction identified in reanalysis of old data in a new sample. Second, by collapsing across these samples before conducting follow-up contrasts we were afforded maximal statistical power to detect the sometimes-subtle effects that underlie this core pattern.

In the remainder of the Methods section we describe the general paradigm adopted in all four experiments before providing details specific to each of the individual experiments.

### General design

Participants viewed visual search arrays consisting of a number of shape outlines presented in a circle formation (see Figure 1). The shapes were unfilled diamonds (4.2°×4.2° visual angle) and circles (1.7° radius) outlined in red or green (0.3° line thickness). Each was presented equidistant from a central fixation point (9.1°) and each other and contained a grey line (0.3°×1.5°) that was randomly oriented to be vertical or horizontal. In every trial one object was a circle with all other objects diamonds; this shape singleton was the target of search and participants were required to report the orientation of the line contained within this object. An additional color singleton was defined in many trials by giving one of the diamonds unique color.

Target and salient distractor locations were randomized with the sole confine that they could not coincide at one location. Each trial began with the presentation of a fixation cross (400 to 1400 ms, rectangular distribution) which was followed by the search array. Correct responses to the search display were immediately followed by a central indication of the number of points acquired in the completed trial, either ‘+1’ or ‘+1’. The magnitude of reward following correct performance was randomly determined for each trial. Incorrect trials resulted in ‘–10’, indicating the loss of 10 points. Feedback was presented to participants for 1000 ms and the search display remained onscreen during the this interval. Participants were instructed to maximize earnings by responding accurately and were paid based on the number of points they accumulated throughout the experiment, but, because reward magnitude was randomly determined and accuracy was high for all participants, there was little variability in pay: no one earned less than 8.00 euro per hour or more than 9.25.

Participants were asked to maintain eye fixation throughout each experimental block. Trials in which response occurred sooner than 100 ms after stimulus onset or later than 2500 ms after were discarded from all analyses (0.8% +/−1.6% of trials, mean +/− SD) and incorrect trials were excluded from calculation of reaction time (RT). Stimuli were presented on a CRT monitor located ∼60 cm from the observer’s eyes. Feedback regarding response latency, average accuracy, and total number of points earned to that point was provided at the end of every block.

For all analyses involving intertrial contingencies the immediately preceding trial had to have occurred in the same block, have been correctly completed, and have involved a search display containing a distractor singleton. Performance in this kind of additional singleton task is substantially more variable in trials where the distractor singleton is present in the display because there is variability in the strength with which this stimulus will capture attention [37]. With this in mind, primary analyses of target location are based on trials where the target was presented in the absence of a salient distractor, with analysis of distractor location necessarily based on trials where both the current and preceding search display contained a salient distractor. To foreshadow, results look much the same if this constraint is not adopted (see Results). In all analyses average per-subject RT reflects the median and average per-subject accuracy reflects the mean.

### Details specific to Experiment 1

Fourteen neurologically typical students of the VU Amsterdam completed this experiment and other analyses of the data formed the basis for a prior report [5]. Participants (21+/−3 years, mean +/− SD; all right handed; 6 women) completed the search task described above where the search array contained 10 shape outlines and the additional color singleton was defined in 75% of trials by giving one of the diamonds unique color, either saturated red while all other objects were saturated green or vice versa. Response was unimanual using the right index and middle fingers on a standard two-button mouse and participants completed 45 blocks of 30 trials. Eye movements were monitored via electrooculogram (EOG). All trials with eye movements identified in an interval beginning 500 ms before stimulus onset and ending 1 s. after were removed from analysis (8+/−4% of trials, mean +/− SD).

### Details specific to Experiment 2

Thirty-seven neurologically typical students of the VU Amsterdam completed this experiment and other analyses of the data formed the basis for a prior report [18]. Data from three participants was removed from analysis due to low accuracy (<2 SD from the mean). Participants (20+/−2 years, mean +/− SD; two left handed; 7 men) completed the search task described above where the search array contained 10 shape outlines and the additional color singleton was defined in 75% of trials by giving one of the diamonds unique color, either saturated red while all other objects were saturated green or vice versa. Response was bimanual, using the left and right index fingers to press the ‘z’ and ‘m’ keys on a standard keyboard, and participants completed 30 blocks of 30 trials.

### Details specific to Experiment 3

Thirty-two neurologically typical students of the VU Amsterdam completed this experiment and other analyses of the data formed the basis for a prior report [19]. Data from two participants was removed from analysis due to low accuracy (<2 SD from the mean). Participants (20+/−2 years, mean +/− SD; 4 left-handed; 11 men) completed a variation of the search task described above where the search array contained 6 shape outlines. For fifteen of these participants the target and homogenous distractors could be characterized by red or green color, with a salient distractor defined in 75% of trials by giving one of the distractors blue color. For the other fifteen this reversed: the target and homogenous distractors were always blue, but a salient distractor was defined in 75% of trials by giving one of the distractors red or green color. Response was bimanual, using the left and right index fingers to press the ‘z’ and ‘m’ keys on a standard keyboard, and participants completed 30 blocks of 30 trials.

### Details specific to Experiment 4

Seventeen neurologically typical students of the VU Amsterdam completed this experiment. In contrast to Experiments 1 through 3, no analysis of this data has been reported elsewhere. Participants (20+/−2 years, mean +/− SD; 4 left-handed; 2 women) completed the search task described above where the search array contained 10 shape outlines and the additional color singleton was defined in 75% of trials by giving one of the diamonds unique color, either saturated red while all other objects were saturated green or vice versa. Response was bimanual, using the left and right index fingers to press the ‘z’ and ‘m’ keys on a standard keyboard, and participants completed 15 blocks of 30 trials.

## Results

Analysis began with consideration of the combined results from Experiments 1, 2 and 3. A RANOVA of RT in this 78-person sample had three factors: *relevant object*, reflecting whether behaviour was binned as a function of the current target location or the current distractor location, *prior location*, reflecting whether the relevant object appeared at the location previously held by a target or distractor, and *prior reward*, reflecting whether high-magnitude or low-magnitude reward was received in the preceding trial (note that trials where neither target nor salient distractor location was repeated were excluded from this analysis). For those subjects who completed the 1.5 hour version of the task the median number of correct trials in the smallest cell of this analysis was 16 trials (13 for 1 hour version). A main effect of relevant object (F(1,77) = 44.68, p<10^−9^, η_p_^2^ = 0.367) in part reflects the presence of the salient distractor: when the target was the relevant item displays did not contain a salient distractor and response was accordingly faster. An interaction between relevant object and prior location (F(1,77) = 33.94, p<10^−7^, η_p_^2^ = 0.306) reflects a speeding when the target reappeared at the target location and slowing when it appeared at the distractor location, but a slowing when the distractor appeared at the target location and speeding when it reappeared at the distractor location. Finally, a critical three-way interaction (F(1,94) = 8.00, p = 0.006, η_p_^2^ = 0.094) indicates that this 2-way pattern varied as a function of reward magnitude in the preceding trial (prior reward×prior location: F(1,94) = 1.01, p = 0.319, η_p_^2^ = 0.013; all other Fs<1). Equivalent analysis of accuracy garnered no significant results (reward: F(1,77) = 1.21, p = 0.274, η_p_^2^ = 0.016; prior location: F(1,77) = 2.01, p = 0.161, η_p_^2^ = 0.025).

Independent analysis of RT from Experiment 4 garnered exactly the same pattern of statistical results. The median number of correct trials in the smallest cell of this analysis was 8. Analysis of this 17-person dataset revealed a main effect of relevant object (F(1,16) = 10.14, p = 0.006, η_p_^2^ = 0.388), an interaction between relevant object and prior location (F(1,16) = 7.13, p = 0.017, η_p_^2^ = 0.308), and a critical 3-way interaction (F(1,16) = 4.59, p = 0.048, η_p_^2^ = 0.223) but no other effects (prior location: F(1,16) = 1.55, p = 0.231, η_p_^2^ = 0.088; reward×prior location: F(1,16) = 2.65, p 0.122, η_p_^2^ = 0.142; reward×relevant object: F(1,16) = 3.10, p = 0.097, η_p_^2^ = 0.162; reward: F<1). Again, equivalent analysis of accuracy garnered no significant results (reward: F(1,16) = 2.13, p = 0.164, η_p_^2^ = 0.118; reward×prior location: F(1,16) = 2.14, p = 0.163, η_p_^2^ = 0.118; all other Fs<1).

**Figure 2:**
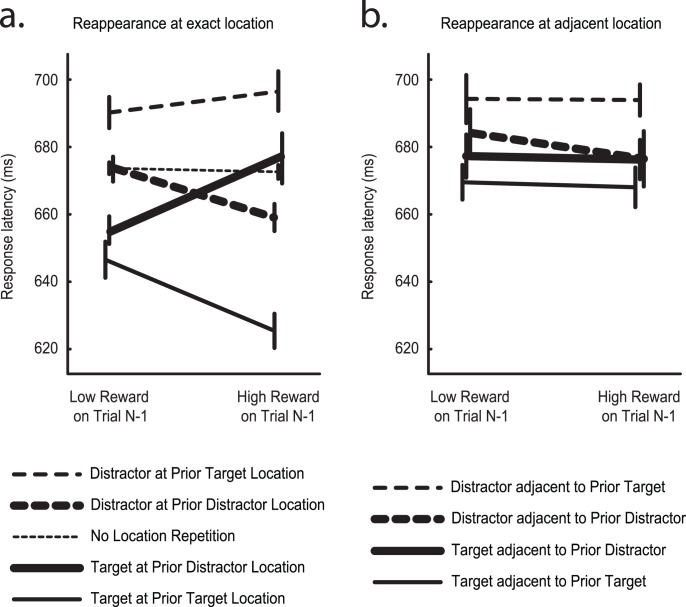
Results from a.) analysis of location repetition, and b.) analysis of reappearance at adjacent location. Error bars here and below reflect within-subject standard error [49].

Results from analysis of the combined data from Experiments 1 through 4 is illustrated in Figure 2a. Planned follow-up tests were conducted on this 95-person dataset. A 2-way RANOVA revealed a significant interaction between prior reward and prior location when analysis was limited to trials where the target or distractor reappeared at the prior distractor location (Figure 2a large trace; interaction: F(1,94) = 7.590, p = 0.007, η_p_^2^ = 0.075; all other Fs<1). A corresponding RANOVA limited to trials where the target or distractor reappeared at the prior target location (Figure 2a small trace) revealed an effect of relevant item (F(1,94) = 71.80, p<10^−12^, η_p_^2^ = 0.433) and an interaction between prior reward and prior location (F(1,94) = 4.74, p = 0.032, η_p_^2^ = 0.048; prior reward: F(1,94) = 2.38, p = 0.126, η_p_^2^ = 0.025). Finally, planned contrasts demonstrated that the effect of reward was reliable when the target reappeared at the target location (Figure 2a small solid trace; t(94) = 2.70, p = 0.008, Cohen’s d = 0.277), when the target reappeared at the distractor location (Figure 2a large solid trace; t(94) = 2.02, p = 0.047, Cohen’s d = 0.207), when the distractor reappeared at the distractor location (Figure 2a large broken trace; t(94) = 2.39, p = 0.019, Cohen’s d = 0.245), but not when the distractor reappeared at the target location (Figure 2a small broken trace; t(94) = 0.70, p = 0.485, Cohen’s d = 0.072), or when neither target or distractor location was repeated (Figure 2a very small broken trace; t(94) = 0.27, p = 0.794, Cohen’s d = 0.027). <footnote 1>.

Consistent with prior findings, the presence of the salient distractor slowed response and decreased accuracy [38], [39] (RT absent: 663 ms, present: 680 ms; t(94) = 8.83, p<10^−7^, Cohen’s d = 0.675; Accuracy: absent: 95.8%, present: 95.4; t(94) = 2.33, p = 0.022, Cohen’s d = 0.239). The magnitude of reward received in the preceding trial had no raw impact on behaviour (RT high-magnitude reward: 670 ms, low-magnitude reward: 671 ms; t(94) = 0.57, p = 0.573, Cohen’s d = 0.059; Accuracy high-magnitude reward: 95.2%, low-magnitude reward: 95.0%; t(94) = 0.85, p = 0.398, Cohen’s d = 0.087).

The 95-person sample includes participants who completed 450, 900, or 1350 trials. During the editorial process a reviewer suggested equating within-subject performance variability across the sample by limiting analysis to only the first 450 trials completed by each participant. This had no impact on the data pattern: an omnibus RANOVA with factors for relevant object, prior location, and prior reward revealed the same three-way interaction (F(1,94) = 8.20, p = 0.005), the same interaction of prior location and relevant object (F(1,64) = 25.28, p<10^−9^), and the same main effect of relevant object (F(1,64) = 18.46, p<10^−5^), but no additional effects (prior reward×prior location: F(1,94) = 2.90, p = 0.092; all other Fs<1).

As noted in the Methods, the analyses detailed above are based on results where target repetition of location was measured in trials where the distractor was absent from the display. The same general pattern of results was observed when this constraint was removed, such that analysis of target repetition was based on all trials. As above, a RANOVA of RT from the 95-person dataset revealed a reliable main effect of relevant object (F(1,94) = 47.74, p<10^−10^, η_p_^2^ = 0.337), an interaction between relevant object and prior location (F(1,94) = 46.73, p<10^−10^, η_p_^2^ = 0.332), and a critical three-way interaction (F(1,94) = 5.58, p = 0.020, η_p_^2^ = 0.056; reward: F(1,16) = 2.31, p = 0.132, η_p_^2^ = 0.024; all other Fs<1).

We conducted an additional analysis to determine the spatial specificity of the effect of reward on location. To this end we examined behaviour when target or distractor reappeared not at the specific locations previously occupied by target or distractor (as detailed above), but rather at the positions immediately adjacent to these locations. If reward has a distributed spatial impact then analysis of hemifield should garner results similar to those detailed above. In contrast, if reward’s effect is spatially constrained, the effect should be larger when analysis is based on specific locations. As is evident in Figure 2b, the pattern illustrated in Figure 2a does not reappear when adjacent locations are considered. A RANOVA analysis of these results with factors for prior reward, prior location, and relevant object revealed a significant interaction between prior location and relevant object (F(1,94) = 12.90, p<0.001; η_p_^2^ = 0.121), apparently driven by a slowing of response when the distractor reappeared close to the prior target location, and a marginal main effect of relevant object (F(1,94) = 3.90, p = 0.051, η_p_^2^ = 0.040; all other Fs<1). Reward had no reliable impact on these results.

We conducted a 4-factor RANOVA in order to contrast results from the two patterns illustrated in Figures 2a and 2b. This had factors for analysis type (same location vs. adjacent location), relevant object, prior location, and prior reward, and revealed a significant four-way interaction (F(1,94) = 7.61, p = 0.007, η_p_^2^ = 0.075). The significant three-way interaction observed when target and distractor reappeared at specific locations was thus reliably different than the far-from-significant pattern observed when they reappeared at adjacent locations. Reward’s impact on locations appears to be strongly circumscribed in space.

Finally, we conducted an exploratory analysis to gain insight into the relationship between reward-priming of location and reward-priming of color. In earlier work with this task we have shown that rewarded target selection will prime subsequent selection of stimuli characterized by the target color. As a result, response is fast and accurate when the target and distractor colors are repeated following high-magnitude reward, but slow and inaccurate when the colors characterizing the target and distractor swap [5], [18]–[19]. The results detailed above additionally demonstrate that high-magnitude reward will prime the spatial location of a target and facilitate suppression of the distractor location. Given that we did not control for this reward-priming of location in our earlier work there is the possibility that reward-priming of color and reward-priming of location interact, with the extreme case being a situation where one of these effects is contingent on the other (as has been suggested of location-priming and feature-priming more generally) [28].

With this in mind we examined the current data as a function of reward history and target color repetition, limiting analysis to trials where the target and salient distractor were presented at locations that had held neither stimulus in the preceding trial. Results from 15 participants were not suited for this analysis because the variant of the experiment completed by these people involved a target that did not change in color (see specific details for Experiment 3 in the Methods section). We accordingly based this analysis on data from the 80 participants who completed a task where the target color was randomly red or green in each trial. For those subjects who completed the 1.5 hour version of the task the median number of correct trials in the smallest cell was 98 trials (64 for 1 hour version, 21 for 1/2 hour version).

**Figure 3:**
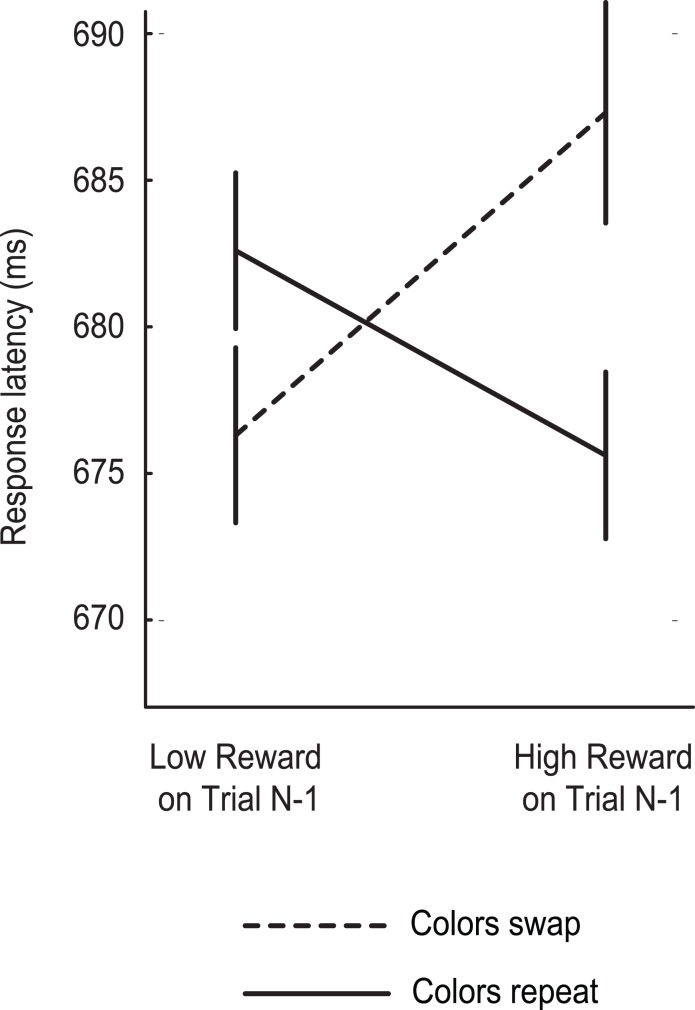
Analysis of color repetition in trials where neither target nor distractor location was repeated.

If reward-priming of color is contingent on reward-priming of location we should find no influence of reward in this analysis. As illustrated in Figure 3, results in fact show an interactive pattern familiar from our earlier work: high-magnitude reward created a performance benefit when the colors were repeated between trials but a cost when the colors swapped (Hickey et al. 2010a). This pattern was reliable in a RANOVA with factors for *prior reward* and *color repetition* (repeat colors vs. swap colors), as reflected in a significant interaction between factors (F(1,79) = 4.56, p = 0.036, η_p_^2^ = 0.055; reward: F(1,79) = 1.14, p = 0.288, η_p_^2^ = 0.014; all other Fs<1). Reward-priming of color thus does not appear contingent on reward-priming of location.

An important caveat must be attached to this last analysis. The data from Experiments 1 through 3 has been used in earlier work to test hypotheses regarding the impact of reward on color priming [5], [18]–[19]. In the primary analyses detailed above we approach this data with new hypotheses regarding the impact of reward on location. However, this last examination of the data - testing if reward-priming of color is contingent on reward-priming of location - was clearly motivated by earlier identification of the color effect in this data. This hypothesis is accordingly *post hoc,* and a core assumption to the use of inferential statistics is not met. Strong conclusions regarding the relationship between reward-priming of color and location will require further dedicated investigation.

## Discussion

The current results demonstrate that location priming in visual search is enhanced by rewarding outcome. We had participants complete a visual search task in which they selected a target, ignored a salient distractor, and received random-magnitude reward for correct performance. High-magnitude reward in one trial facilitated the return of attention to the target position and inhibited the deployment of attention to the location that had held the salient distractor. As a result, we observed a behavioural benefit following reward when the target or distractor location was repeated, but an exacerbated cost when the target appeared at the former distractor location. This pattern suggests that reward outcome guides the manner in which humans deploy attention through space.

Importantly, the priming indexed in the current data does not appear strategic in nature. Target and distractor locations in the experimental design were random. This feature of the design would have become apparent to participants after a handful of experimental trials and meant that there was no motivation for them to establish a top-down, strategic attentional set for any particular location in space. We believe that the results rather reflect low-level plasticity in visual representation. Recent models of visual learning suggest that such plasticity may occur when a.) attention is applied to a stimulus, and b.) there is concurrent release of a diffuse neuromodulatory signal in visual cortex signalling the receipt of unexpected reward [40]–[41]. When participants in the current study attended the target and were rewarded for doing so, the resulting reward-elicited neuromodulatory signal may have automatically reinforced the cognitive ‘act’ of enhancing processing at the target location and inhibiting processing at the location of the salient distractor.

A developing literature supports the notion that this kind of plasticity can occur in the absence of volition, strategy, or even awareness. For example, imaging results have shown that reward-associated stimuli will evoke increased activity in visual cortex even when participants are unaware that a stimulus was presented [42]. Participants will learn about stimuli paired with reward when these stimuli are rendered nonconscious through continuous flash suppression [43] or gaze-contingent crowding [44], and reward-associated stimuli will preferentially ‘break through’ such procedures to reach awareness. Consistent with the idea that plasticity may in part rely on selective attention, recent results have demonstrated that factors impacting attentional selection - like perceptual grouping - also have clear effects on perceptual learning [45].

Our interpretation of the results is evocative of instrumental learning accounts of overt behaviour. Instrumental learning is traditionally characterized by an observable change in external action, as when an animal is gradually trained to press a lever by rewarding behaviour that brings it closer to this goal state. However, accumulating research suggests that the tenets of instrumental learning may also be important to our understanding of the activation of covert cognitive mechanisms [4]. By this, the action of such mechanisms is reinforced by good outcome, increasing the likelihood that they be deployed under similar circumstances in the future. In the context of the current data, we believe that rewarding outcome acted to prime both mechanisms that enhance the representation of stimuli at a specific location and those that suppress the representation of stimuli at nontarget locations [35]–[36]. This priming has a carryover impact on performance in the next trial such that spatial selection became biased toward stimuli at the former target location and away from stimuli at the former distractor location.

In the current results both positive and negative priming effects were spatially specific, emerging only when the target and distractor stimuli appear at the discrete locations that had contained one of these stimuli in the preceding trial (see Figure 2). This is in contrast to a prior study of location priming in search from Kumada and Humphreys [31], where positive priming effects were found to have the same specificity observed in the current data, but negative priming effects were of much the same magnitude regardless of whether the target appeared at the specific location that formerly held the distractor or somewhere in the same visual hemifield. This incongruity between studies may stem from a small change in experimental design. In the paradigm used by Kumada and Humphreys [31] the target and salient distractor could be presented at only four possible locations, two on each side of the display, and when the distractor was present in the display it was always in the hemifield contralateral to the target. This was not the case in our design, where the target and salient distractor locations were unconstrained. This meant that the stimuli could appear in the same hemfield, and even in adjacent positions, likely creating the need for a more spatially-specific application of attention to resolve target information. If the attentional mechanisms responsible for target enhancement and distractor suppression acted with tighter focus it is reasonable that their residual effects are also more spatially constrained.

Prior analysis of the current data has shown a.) that reward will speed target response when the colors characterizing the target and salient distractor are repeated between trials, but b.) that reward will slow response when these colors swap [5]. In the results section above we detail an exploratory analysis suggesting that this reward-priming of color is independent of the reward-priming of location that is the primary topic of the current paper (see Figure 3). This suggests that reward-priming of location is not contingent on reward-priming of color (as has been suggested of location priming and feature priming more generally) [28], [46]. However, our expectation is that these effects ultimately reflect action of attentional mechanisms that will commonly be activated under the same circumstances and that they should accordingly covary to a large degree.

We have suggested elsewhere that reward-priming of color might reflect a low-level mechanism with evolutionary origins [5], [9]. According to this idea, reward signals encoded in mesolimbic dopamine act to bias perception and attention towards objects that have acted as valid reward cues in the past [47]–[48]. The current results suggest that this general function is created through the action of at least two mechanisms, one working on the visual features that characterize relevant and irrelevant stimuli, the other acting on the contextual location of such stimuli. Because both objects and locations that have proven beneficial in the past are likely to prove beneficial in the future these reward-priming mechanisms could provide very real evolutionary utility.
